# Heme is involved in the exogenous ALA-promoted growth and antioxidant defense system of cucumber seedlings under salt stress

**DOI:** 10.1186/s12870-022-03717-3

**Published:** 2022-07-08

**Authors:** Yue Wu, Jing Li, Junwen Wang, Mohammed Mujitaba Dawuda, Weibiao Liao, Xin Meng, Hong Yuan, Jianming Xie, Zhongqi Tang, Jian Lyu, Jihua Yu

**Affiliations:** 1 College of Horticulture, Gansu Agricultural University, Lanzhou, 730070 China; 2 Department of Horticulture, University for Development Studies, Tamale, Ghana; 3 State Key Laboratory of Arid-Land Crop Science, Gansu Agricultural University, Lanzhou, 730070 China

**Keywords:** Salt stress, Cucumber, 5-aminolevulinic acid, Heme, Anti-oxidant defense system

## Abstract

A biosynthetic precursor of tetrapyrrol, 5-aminolevulinic acid (ALA), is widely used in agricultural production, as an exogenous regulatory substance that effectively regulates plant growth. Previous studies have shown that heme and chlorophyll accumulate in plants under salt stress, when treated with exogenous ALA. In this study, we explored the regulatory role of heme in plants, by spraying 25 mg L^–1^ ALA onto the leaves of cucumber seedlings treated with heme synthesis inhibitor (2,2'-dipyridyl, DPD) and heme scavenger (hemopexin, Hx), under 50 mmol L^–1^ NaCl stress. The results showed that NaCl alone and DPD + Hx treatments to cucumber seedlings subjected to salt stress adversely affected their growth, by decreasing biomass accumulation, root activity, and root morphology. In addition, these treatments induced an increase in membrane lipid oxidation, as well as enhancement of anti-oxidase activities, proline content, and glutamate betaine. However, exogenous ALA application increased the plant growth and root architecture indices under NaCl stress, owing to a lack of heme in the seedlings. In addition, cucumber seedlings treated with DPD and Hx showed inhibition of growth under salt stress, but exogenous ALA effectively improved cucumber seedling growth as well as the physiological characteristics; moreover, the regulation of ALA in plants was weakened when heme synthesis was inhibited. Heme biosynthesis and metabolism genes, *HEMH* and *HO1*, which are involved in the ALA metabolic pathway, were upregulated under salinity conditions, when ferrochelatase activity was inhibited. Application of exogenous ALA increased the heme content in the leaves. Thus, exogenous ALA may supplement the substrates for heme synthesis. These results indicated that heme plays a vital role in the response of plants to salinity stress. In conclusion, heme is involved in ALA-mediated alleviation of damage caused to cucumber seedlings and acts as a positive regulator of plant adaption.

## Introduction

In recent times, deteriorating natural climates and increasing human activity have seriously threatened the development of agriculture across the globe. Crop growth suffers from environmental stresses, such as high or low temperature [1, 2], drought [3] and high salinity [4]. These abiotic stresses not only directly affect the growth and development of plants, but also decrease their yield and economic benefits [5]. Globally, salt stress is a prevalent abiotic stress that inhibits plant growth [6]. When plants are exposed to saline conditions, high concentrations of sodium (Na^+^) and chloride (Cl^−^) affect many of their physiological and biochemical processes/functions. For example, the biosynthesis of chlorophyll and photosynthesis reactions can be inhibited under salt stress [7], in addition to the water and nutrient absorption of the root system [8].

Salt stress inhibits the normal growth of plants, mainly through osmotic, ion, and oxidative stress. The homeostasis of production and elimination of reactive oxygen species (ROS) [9] in plants is damaged by excessive concentrations of Na^+^ and Cl^−^ ions [10, 11]. Plants have a well-developed and powerful antioxidant protection system, which mainly includes antioxidant enzymes and osmotic substances. For example, catalase (CAT), superoxide dismutase (SOD), peroxidase (POD), and ascorbate peroxidase (APX) can reduce the levels of ROS [9]. In addition, glycine betaine and proline also regulate the osmotic balance in plants [12]. However, under salinity stress, plants need to improve their anti-oxidant capacity, to adapt to stressful conditions.

Plant growth regulators play important roles in plant physiology and metabolism, and are often used to enhance the stress resistance of plants. A relatively new plant growth regulator known as 5-aminolevulinic acid (ALA) alleviates the harmful effects of abiotic stress in plants, by enhancing photosynthetic efficiency, improving mineral nutrient absorption, increasing water transport, and maintaining anti-oxidant enzyme activities [13–15]. ALA is a metabolic intermediate that is commonly found in plants, fungi, and bacteria [16]. In higher plants, it serves as a precursor of chlorophyll and heme, as well as that of all tetrapyrrole substances (including siro-heme, vitamin B_12_, and phytochrome) [17]. To date, a lot of studies have shown that ALA plays a role in alleviating salt stress in plants such as *Solanum lycopersicum*, *Citrullus lanatus* (Thunb.) Matsum., and Nakai [18–21].

Many studies have demonstrated that exogenous ALA increases the content of endogenous products, including chlorophyll and heme, in plants under salt stress [22–24]. Increasing levels of chlorophyll leads to an enhanced photosynthesis defense system and accumulation of organic matter in plants [20, 25]. Heme is a major co-factor that is involved in the transformation of superoxide anions in the anti-oxidant system. It has been shown that the heme content of plants is related to plant tolerance, and salt stress could greatly reduce the endogenous heme content of plants [26, 27]. Exogenous ALA increased the accumulation of heme in plants and alleviated the injury caused by water deficit and salt stress [15, 28, 29]. In the current study, we hypothesized that an increase in heme content might be related to the adaptability of plants to stress.

Few studies have indicated that heme has strong photodynamic cytotoxicity, and its production could be associated with an increase in ROS, which subsequently causes damage to the cells. Moreover, pigment sensing proteins (TPSO) in plants may be involved in oxidative stress tolerance, by combining and eliminating free heme [30, 31]. However, other researchers have an opposing view; they found that the addition of heme to *Arabidopsis* did not induce the production of ROS, and heme is a signal transduction conductor from chloroplast to cytoplasm, as well as, a factor essential for plant tolerance [32, 33]. Overexpression of the iron chelate gene (*BjFeCh*) of *Bradyrhizobium japonicum* in rice increased the amount of heme and the resistance of transgenic rice to herbicide stress [34]. Therefore, based on the above two viewpoints, it is particularly important to study whether the response of heme in plants to environmental stress is positive or negative.

To investigate the role of heme synthesis in plant abiotic stress, we combined a heme scavenger and a ferrochelatase (FECH) synthetic inhibitor, to inhibit the heme biosynthesis pathway in plants for the first time. Currently, two inhibitors and one scavenger are widely used in medical science. N-methylmesoporphyrin (N-MMP) is a competitive inhibitor of FECH [35]. *Plasmodium falciparum*, a parasite dependent on FECH activity, can be inhibited by applying N-MMP in vitro [36]. Another FECH inhibitor is 2,2'-dipyridyl (DPD). In plant tissues, DPD can instantaneously chelate ferrous ions (Fe^2+^) to produce terpyridine-Fe compounds. Therefore, FECH loses its substrate and against normally heme synthesize [37]. The heme scavenger hemopexin (Hx), also known as thrombin, has a high affinity for free heme and can bind with heme to form the Hx-Heme complex. It has been proven in blood studies on mice that Hx can treat hemolytic disease in mice and clear the excess free heme [38, 39]. In the present study, cucumber seedlings were treated with heme inhibitors and scavengers. In addition, biochemical and molecular indices were determined upon application of exogenous ALA and rhizospheric NaCl.

## Materials and methods

### Plant material and growth conditions

Cucumber (*Cucumis sativus* L. cv. Xinchun No. 4) seeds of uniform size were dipped in distilled water for 6 h, surface-disinfected with 0.03% KMnO_4_ for 15 min, and rinsed with distilled water. The seeds were germinated on wet filter paper, under dark conditions in a growth chamber (28 ± 1 °C). At five days after germination, the seedlings with fully spread cotyledons and well-grown roots were transplanted into plastic boxes (volume 800 mL) and then put into a climate chamber (temperature 18/28 °C, photoperiod 12 h/12 h, light intensity 350–450 μmol m^–2^ s^–1^, and humidity 60%–70%). Two plants were placed in each box. A hydroponic nutrient solution was prepared as described by Yamasaki (1981), and its 1/3 dilution was used to grow cucumber seedlings, with the nutrient solution changed at 2-d intervals.

### Experimental design

#### Experiment I

Twenty-day-old seedlings with two true leaves were used to select the optimum chemical concentrations (including those for NaCl, ALA, Hx, DPD, and N-MMP). The NaCl concentration that could cause moderate salt stress was also identified. The NaCl concentrations tested included 0, 10, 25, 50, and 75 mmol L^–1^. NaCl was added to the Yamazaki cucumber nutrient solution, and the treatments were applied and monitored for seven days. By observing the morphological characteristics and measuring the growth parameters of the cucumber seedlings, it was found that 50 mmol L^–1^ caused moderate salt stress in them.

The most effective ALA concentration for mitigating salt stress (50 mmol L^–1^) in cucumber seedlings was determined. ALA concentrations of 0, 10, 25, 50, and 75 mg L^–1^ under 50 mmol L^–1^ NaCl stress were used. Both surfaces of the leaves were uniformly sprayed with ALA (Sigma-Aldrich, USA) solution using a hand-held sprayer in the dark, following which the appropriate ALA concentration was chosen by considering the morphological and growth indices of the plants. We found that 25 mg L^–1^ ALA could alleviate moderate salt stress, and thus, this concentration was used in subsequent experiments.

Appropriate concentrations of heme scavengers (Hx) and heme biosynthesis inhibitors (DPD and N-MMP) that could suppress the heme branch in the ALA metabolism pathway were selected. Different concentrations of Hx, DPD, and N-MMP were sprayed onto the leaves and monitored for seven days. Moreover, the minimum content of endogenous heme in plant leaves without apparent damage was considered the screening standard. Hx concentrations of 0, 0.2, 0.4, 0.8, and 1.6 μg L^–1^ were tested. Consequently, 0.4 μg L^–1^ Hx was found to have the greatest restraining effect on heme biosynthesis in cucumber seedlings, which was then used in the further studies. Analogously, the DPD concentrations were set to 0, 0.1, 0.2, 0.4, and 0.8 mmol L^–1^. After assessment of plant morphology and endogenous heme content, 0.2 mmol L^–1^ DPD was selected as the most effective concentration for inhibiting heme biosynthesis in cucumber seedlings. The N-MMP concentrations tested included 0, 0.2, 0.4, 0.8, and 1.6 μg L^–1^. None of the treatments had a significant effect on the endogenous heme content in the cucumber leaves; therefore, they were not chosen for further testing.

#### Experiment II

After screening the concentrations of NaCl, ALA, Hx, and DPD, the different treatments of the experiment were decided as the following: (1) control: normal growth condition; (2) N: 50 mmol L^–1^ NaCl in nutrient solution; (3) A: foliar-applied 25 mg L^–1^ ALA; (4) NA: 50 mmol L^–1^ NaCl + 25 mg L^–1^ ALA; (5) NHD: 0.4 μg L^–1^ Hx + 0.2 mmol L^–1^ DPD + 50 mmol L^–1^ NaCl; (6) NAHD: 50 mmol L^–1^ NaCl + 25 mg L^–1^ ALA + 0.4 μg L^–1^ Hx + 0.2 mmol L^–1^ DPD; (7) HD: 0.4 μg L^–1^ Hx + 0.2 mmol L^–1^ DPD. ALA was applied 12 h after Hx and DPD administration. Each replicate contained 12 plastic containers (volume 600 mL), and each treatment was replicated three times. The containers were arranged in a completely randomized order in a growth chamber. The treatments were executed every day and lasted for 7 d.

### Cucumber growth indices

Plant height, stem diameter, dry weight, and fresh weight of the cucumber seedlings were measured to determine the appropriate chemical (NaCl and ALA) concentrations for Experiment I, as well as the differences among the different treatments in Experiment II. In addition, the morphology of the seedlings was observed using a digital camera.

### Contents of derivatives of the ALA metabolic pathway

Endogenous ALA content was measured according to the method of Morton, with some modifications [40]. Fresh leaf samples (5 g) were homogenized in an acetate buffer (pH 4.6) on ice. The homogenate was subjected to centrifugation for 15 min at 5000 g, 4 °C. Acetylacetic ester was added to the supernatant and incubated at 100 °C, in a water bath for 10 min. When the mixture cooled to room temperature, an equal volume of fresh Ehrlich’s reagent solution was added, and the reaction was performed for 15 min. The optical density values were measured at the wavelength of 554 nm and the ALA concentration was calculated using an ALA standard curve.

The Proto IX and Mg-Proto IX contents were determined according to the method of Hodgins and Van Huystee, with some modifications [41]. Fresh leaf samples were homogenized with 80% alkaline acetone, and the volume was adjusted to 25 mL with 80% alkaline acetone. The mixture was kept in the dark until the leaf tissue was bleached. The extract was then centrifuged at 1500 g for 10 min. The absorbance of the supernatant was measured at the wavelengths of 575 nm, 590 nm, and 628 nm. The derivative contents were calculated using the corresponding formulae [42]:

$$ProtoIX\;(\mu mol\:g\:FW-1)\:=\:0.18016\:\times\:A575\:-\:0.04036\:\times\:A628\:-\:0.04515\:\times\:A590\:\times\:V\:/\:FW.$$

$$Mg-ProtoIX\;(\mu mol\:g\:FW-1)\:=\:0.06077\:\times\:A590\:-\:0.01937\:\times\:A575\:-\:0.003423\:\times\:A628\:\times\:V\:/\:FW.$$

where V is the dissolved volume of the determined solution and FW is the weight of the fresh sample.

Heme content in the cucumber leaves was determined according to our previous method [24]. The fresh leaf sample (2 g) was ground in liquid nitrogen, and then mixed with 5 mL of extract I (0.5 mL of 0.1 mol L^–1^ ammonia and 4.5 mL pure acetone). The mixture was subjected to centrifugation at 8000 g for 10 min. This process was repeated until the chlorophyll was completely removed. Next, 5 mL of extract II (80% acetone, 16% dimethyl sulfoxide, and 4% concentrated sulfuric acid) was added to the sediment and then subjected to centrifugation at 8000 g for 10 min. Following that, 0.7 mL ethanol was mixed with the supernatant. The absorbance of the mixture was measured at the wavelength of 386 nm. Heme concentration was calculated using a standard curve of heme reference standards.

### Root morphological characteristics

Morphological characteristics of cucumber roots, including total root length, root tip number, root surface area, and root volume, were determined using a root scanner (STD 4800, Canada) and analyzed using WinRHIZO™ 5.0 (Regent Instruments Inc., Canada).

### Root activity

Root activity of cucumber seedlings was determined using triphenyl tetrazolium chloride (TTC), according to the method of Lindström [43]. Cucumber root sample (0.5 g) was soaked in 10 mL mixture solution [containing 5 mL 0.4% TTC and 5 mL phosphate buffer solution (pH 7.0)], following which the samples were placed in the dark, at 37 °C for 1 h. Subsequently, 2 mL of 1 mol L^–1^ sulfuric acid was added to stop the reaction. The root sample was removed, wiped with filter paper, and ground with 3–4 mL ethyl acetate, to extract the red insoluble matter. The red extract solution was transferred to a test tube, following which the residue was washed two or three times with 1 mL of ethyl acetate. Ethyl acetate was then added to make up the total volume to 10 mL. Finally, the absorbance was measured at the wavelength of 485 nm, and the concentration of root activity was calculated using the TTC standard curve.

### Membrane permeability

Membrane permeability was determined using fresh leaves of cucumber seedlings, according to the method described by Inal [44]. The leaf sample (10 g) was cut and put into a conical flask, following which 15 mL distilled water was added to it, steeped, and placed at 25 ± 1 °C for 30 min. The original electrical conductivity (EC1) was measured using a conductometer (Shanghai Yidian Scientific Instrument Co. Ltd., China) and the electrical conductivity (EC2) of the sample was measured after 30 min in a hot water bath. The membrane permeability was calculated according to the formula:

$$\mathrm{EL}\:=\:(\mathrm{EC}1/\mathrm{EC}2)\:\times\:100\%.$$

### Anti-oxidant enzyme activity

Cucumber leaf samples (0.1 g) were ground with 1 mL extracting solution in an ice bath, following which the homogenate was subjected to centrifugation for 10 min, at 8000 g, 4 °C. The supernatant was collected, and subsequent determination was performed according to the manufacturer’s instructions. The activities of SOD, CAT, POD, and APX were determined using enzyme activity kits (Suzhou Keming Biotechnology Co. Ltd., China).

### Measurement of the contents of osmotic adjustment substances

A fresh cucumber leaf sample was used to measure the proline content. Leaf samples (0.1 g) were ground with 1 mL extracting solution in an ice bath after thorough incorporation, and then extracted in a water bath, at 95 °C for 10 min. The homogenate was then subjected to centrifugation for 10 min, at 10,000 g, 25 °C. The obtained supernatant was collected, and subsequent operations were performed according to the manufacturer’s instructions. The proline content was measured using a kit (Keming Biotechnology Co. Ltd., China), according to the manufacturer’s instructions.

A dry leaf sample of cucumber was used to determine the glycine betaine content. The sample (0.04 g) was fully ground, passed through a 40-mesh sieve, following which 1.6 mL of water was added to it. The mixture was placed at 60 °C for extraction, for 30 min, following which 400 μL extracting solution was added to it, mixed, and the mixture was subjected to centrifugation for 10 min, at 10,000 g, 25 °C. The supernatant was used for determination. Glycine betaine content was measured using a kit (Keming Biotechnology Co. Ltd.), according to the manufacturer’s instructions.

Fresh cucumber leaf samples were used to measure the glucose content. Fresh leaves (0.1 g) were placed in a mortar containing 1 mL of distilled water and ground into a homogenate. The homogenate was then incubated in a water bath, at 95 °C for 10 min. After cooling, the homogenate was subjected to centrifugation for 10 min, at 8000 g, 25 °C. The supernatant was used for determination. The glucose content was measured using a kit (Keming Biotechnology Co. Ltd.), according to the manufacturer’s instructions.

The starch content was determined using fresh cucumber leaf samples. A fresh sample (0.1 g) was placed in a mortar with 1 mL of extracting solution I from the kit and fully homogenized. Subsequently, it was incubated in a water bath, at 80 °C for 30 min. After cooling, the homogenate was subjected to centrifugation for 10 min at 3000 g, 25 °C. Following that, the sediment was collected, 0.5 mL distilled water was added to it, and it was gelatinized in a water bath, at 95 °C for 15 min. Thereafter, 0.35 mL of extracting solution II from the kit was added to the mix, and incubated in a water bath, at 25 °C for 15 min. Finally, 0.85 mL distilled water was added to the mix, fully mixed, and subjected to centrifugation for 10 min at 3000 g, 25 °C. The supernatant obtained was used for determination. The starch content was measured using a kit (Keming Biotechnology Co. Ltd.) according to the manufacturer’s instructions.

### Expression levels of the key genes involved in the ALA metabolic pathway

The gene transcriptional levels of *HEMA1* (which encodes glutamyl-tRNA reductase), *CHLH* (which encodes the H subunit of Mg-chelatase), *HEMH* (which encodes ferrochelatase), *GUN4* (a co-factor of Mg-chelatase), and *HO1* (which encodes heme oxygenase 1) were determined using qPCR. Total RNA was extracted from cucumber leaf samples using a plant RNA extraction kit (Tiangen Biotech, China). Reverse transcription of cDNA was conducted using the FastKing RT Kit (Tiangen Biotech). The cucumber *GAPDH* gene was used as an internal control. The GenBank accession numbers and primers used in this study are listed in Table 1. PCR was conducted using a real-time PCR detection system (QuantStudio™ 5 Real-Time PCR System, Thermo Fisher Scientific, USA). The qPCR reaction consisted of 2 μL cDNA, 7.5 μL 2 × qPCR mix, 1.5 μL forward primer (2.5 μmol L^–1^), 1.5 μL reverse primer (2.5 μmol L^–1^), and RNase-free ddH_2_O to make up final volume to 20 μL. The PCR procedure was executed with three technical replicates per biological sample. The PCR reaction conditions were: pre-denaturation at 95 °C for 10 min, followed by 40 cycles of denaturation at 95 °C for 15 s, annealing and extension at 60 °C for 30 s. Finally, the relative expression level for each sample as compared to that of the control was calculated using the 2^−ΔΔCT^ method [45].

**Table 1:** Primer sequences and GenBank accession numbers for *HEMA1*, *CHLH*, *HEMH*, *GUN4*, *HO1*, and *GAPDH* genes

Gene symbol	Accession number	Forward primer	Reverse primer
*HEMA1*	NM001280574.1	CGGTGGGTAAACGGGTAAGAACAG	ATTCTGGCAGTGGCATGTGAAGG
*CHLH*	XM004149349.3	CCTGATAACACCAACGGCCTTCC	CTTGTACGGCGGCTGTGAGTG
*HEMH*	NM001308874.1	GAAGCCCTTCCATCCGCAACG	AAGATGAACGCAAGGACCGAACC
*GUN4*	XM004140838.3	TCACCACCACCACCACCACTAC	GCGGAGGAGGTGTCTGAGGAG
*HO1*	HQ198046.1	GAGGAGATGAGGTTTGTGGCGATG	GTCGGCTCCCATTTAGCAACAGG
*GAPDH*	NM001305758.1	AGTAGCCAAACCATCTATTCAGGC	CAAGTGGGGAATCCTTGCGT

### Statistical analysis

Data were collected and subjected to analysis of variance. The results are presented as mean ± standard error. Mean separation was performed using Tukey’s test, at a probability level of 0.05, using SPSS software (version 22.0; IBM, USA). The figures were prepared using OriginPro2017 (OriginLab Institute Inc., USA).

## Results

### Effect of different concentrations of NaCl on cucumber seedlings

After treatment with different concentrations of NaCl for 7 d, the plant height, stem diameter, fresh weight, and dry weight of the cucumber seedlings first increased and then decreased with increasing NaCl concentration (Table 2). Plant height, stem diameter, fresh weight, and dry weight of seedlings increased significantly under the 10 mmol L^–1^ NaCl treatment. At higher NaCl concentrations (50 and 75 mmol L^–1^), the growth of the cucumber seedlings was significantly inhibited. Based on the effects of different concentrations of NaCl on plant height, stem diameter, plant dry weight, and fresh weight of cucumber seedlings, 10 mmol L^–1^ NaCl was considered as the stimulative concentration, 25 mmol L^–1^ NaCl as the mild salt stress concentration, 50 mmol L^–1^ NaCl as the moderate salt stress concentration, and 75 mmol L^−1^ NaCl as the severe salt stress concentration. Based on this, 50 mmol L^–1^ NaCl, which caused moderate salt stress, was used in the subsequent experiments.

**Table 2:** Growth indices of cucumber seedlings under different NaCl stresses

NaCl concentrations (mmol L^–1^)	Plant height (cm)	Stem diameter (mm)	Fresh weight (g plant^–1^)	Dry weight (g plant^–1^)
0	9.30 ± 0.40 b	4.59 ± 0.46 ab	10.74 ± 0.41 ab	0.63 ± 0.03 b
10	10.40 ± 0.76 a	5.43 ± 0.15 a	12.08 ± 0.30 a	0.89 ± 0.04 a
25	8.32 ± 0.91 ab	4.46 ± 0.21 b	9.49 ± 0.63 bc	0.56 ± 0.03 c
50	6.97 ± 0.37 c	4.32 ± 0.15 b	8.85 ± 0.50 b	0.59 ± 0.04 c
75	5.98 ± 0.20 c	3.88 ± 0.15 c	7.78 ± 0.49 c	0.34 ± 0.03 d

Values are presented as mean ± SE (*n* = 6). Different letters in each column indicate significant differences under the different NaCl stress treatments (*P* < 0.05)

### Effect of different concentrations of ALA on cucumber seedlings

Different concentrations of ALA were sprayed on the leaves of cucumber seedlings exposed to 50 mmol L^–1^ salt stress. Plant height, stem diameter, fresh weight, and dry weight of cucumber seedlings were measured seven days after treatment (Table 3). Application of 10 mg L^–1^ ALA increased the plant height, stem diameter, fresh weight, and dry weight of the cucumber seedlings under 50 mmol L^–1^ NaCl stress. However, 25 mg L^–1^ ALA displayed the greatest mitigating effect, and increased the cucumber plant height by 48.85%, the stem diameter by 12.70%, the fresh weight by 32.17%, and the dry weight by 25.0%. In addition, 50 and 75 mg L^–1^ ALA significantly decreased the growth and biomass of the cucumber seedlings. Therefore, we selected 25 mg L^–1^ ALA as the most appropriate concentration to alleviate the adverse effects of 50 mmol^–1^ salt stress, and used it in subsequent experiments.

**Table 3:** Growth indices of the cucumber seedlings subjected to different ALA concentrations under medium NaCl stress

ALA concentration (mg L^–1^)	Plant height (cm)	Stem diameter (mm)	Fresh weight (g plant^–1^)	Dry weight (g plant^–1^)
0	6.98 ± 0.08 c	4.62 ± 0.07 c	9.17 ± 0.04 c	0.52 ± 0.01 b
10	8.75 ± 0.15 b	5.01 ± 0.7 ab	10.09 ± 0.08 b	0.55 ± 0.02 ab
25	10.39 ± 0.23 a	5.21 ± 0.05 a	12.12 ± 0.07 a	0.65 ± 0.01 a
50	6.87 ± 0.1 c	3.98 ± 0.04 d	9.16 ± 0.04 c	0.49 ± 0.01 c
75	5.62 ± 0.09 d	3.71 ± 0.06 e	8.86 ± 0.03 a	0.37 ± 0.01 d

Values are presented as mean ± SE (*n* = 6). Different letters in each column indicate that ALA treatments at different concentrations displayed significantly different effects under 50 mmol L^–1^ NaCl stress (*P* < 0.05)

### Concentration screening of appropriate FECH inhibitors (DPD and N-MMP) and heme s cavengers (Hx)

**Fig. 1:**
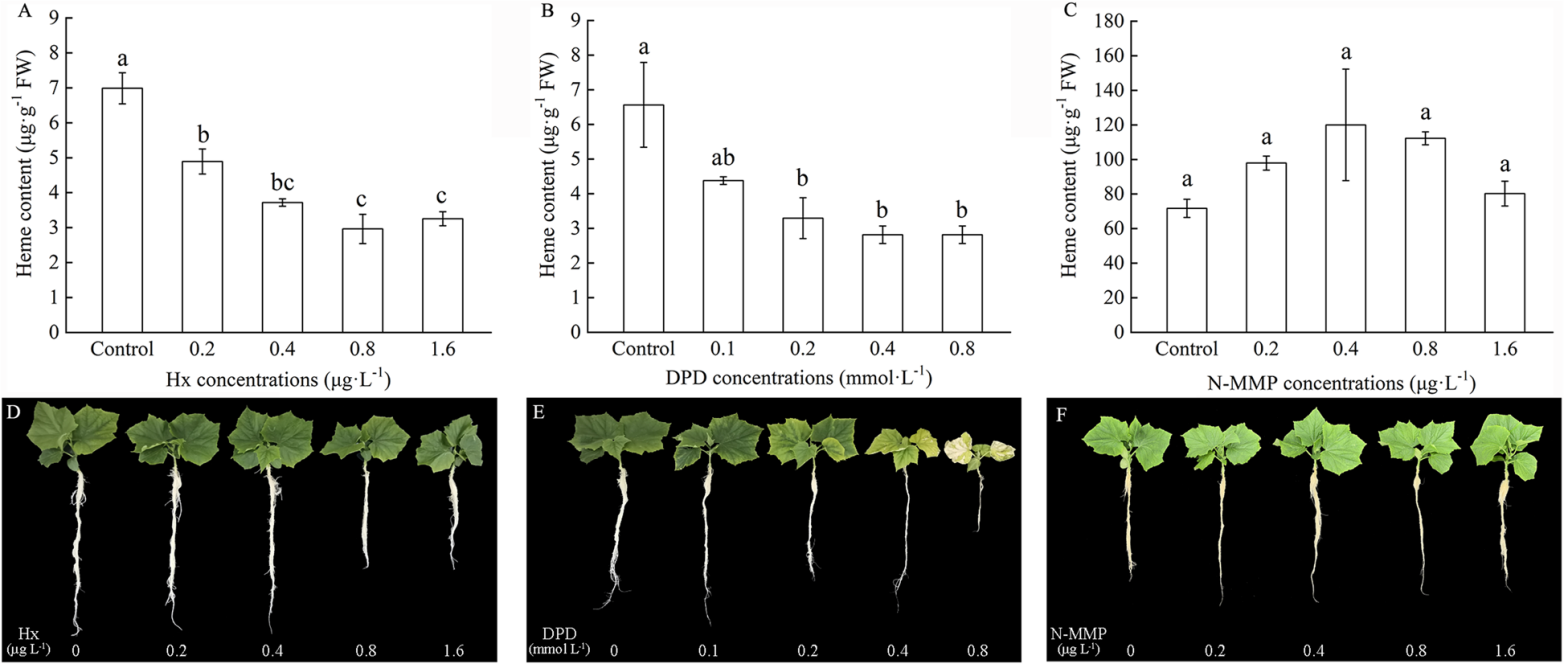
Effects of different concentrations of Hx, DPD and N-MMP on the endogenous heme content and morphology of cucumber seedlings. **A** Endogenous heme content of cucumber seedlings under different concentrations of Hx. **B** Endogenous heme content of cucumber seedlings under different concentrations of DPD. **C** Endogenous heme content of cucumber seedlings under different concentrations of N-MMP. **D** Photos of plant morphology selected by Hx concentration. **E** Photographs of plant morphology screened by DPD concentration. **F** Photographs of plant morphology screened by N-MMP concentration. Values were mean ± SE (*n* = 3). Bars with different lowercase letters were significantly different by Tukey’s multiple range test (*P* < 0.05)

The endogenous heme content of cucumber seedlings treated with different concentrations of DPD, N-MMP, and Hx is shown in Fig. 1. With an increase in DPD and Hx content, the heme content in cucumber leaves gradually decreased. Compared to that in the control, the endogenous heme content in the cucumber leaves decreased slightly under 0.1 mmol L^–1^ DPD treatment, but underwent significant decreases of 49.8%, 57.1%, and 57.1% with DPD concentrations of 0.2, 0.4, and 0.8 mmol L^–1^, respectively. However, the morphology of the seedling was observably altered by 0.4 mmol L^–1^ DPD; upon treatment with 0.8 mmol L^–1^ DPD, the seedling got bleached and died. The endogenous heme content in the cucumber leaves was significantly reduced under 0.2, 0.4, 0.8, and 1.6 μg L^–1^ Hx, by 29.9%, 46.8%, 57.6%, and 53.4%, respectively. Seedling growth morphology was observably inhibited by 0.8 and 1.6 μg L^–1^ Hx. Moreover, when the concentration of N-MMP increased, the heme content increased slightly. In addition, none of the N-MMP treatments significantly affected the heme content or plant morphology. By considering the content of endogenous heme in cucumber leaves as well as the maintenance of relative normal growth of plants, we selected 0.2 mmol L^–1^ DPD and 0.4 μg L^–1^ Hx as the concentration for treatment in further experiments.

### ALA increased the biomass of cucumber seedlings under NaCl stress

**Fig. 2:**
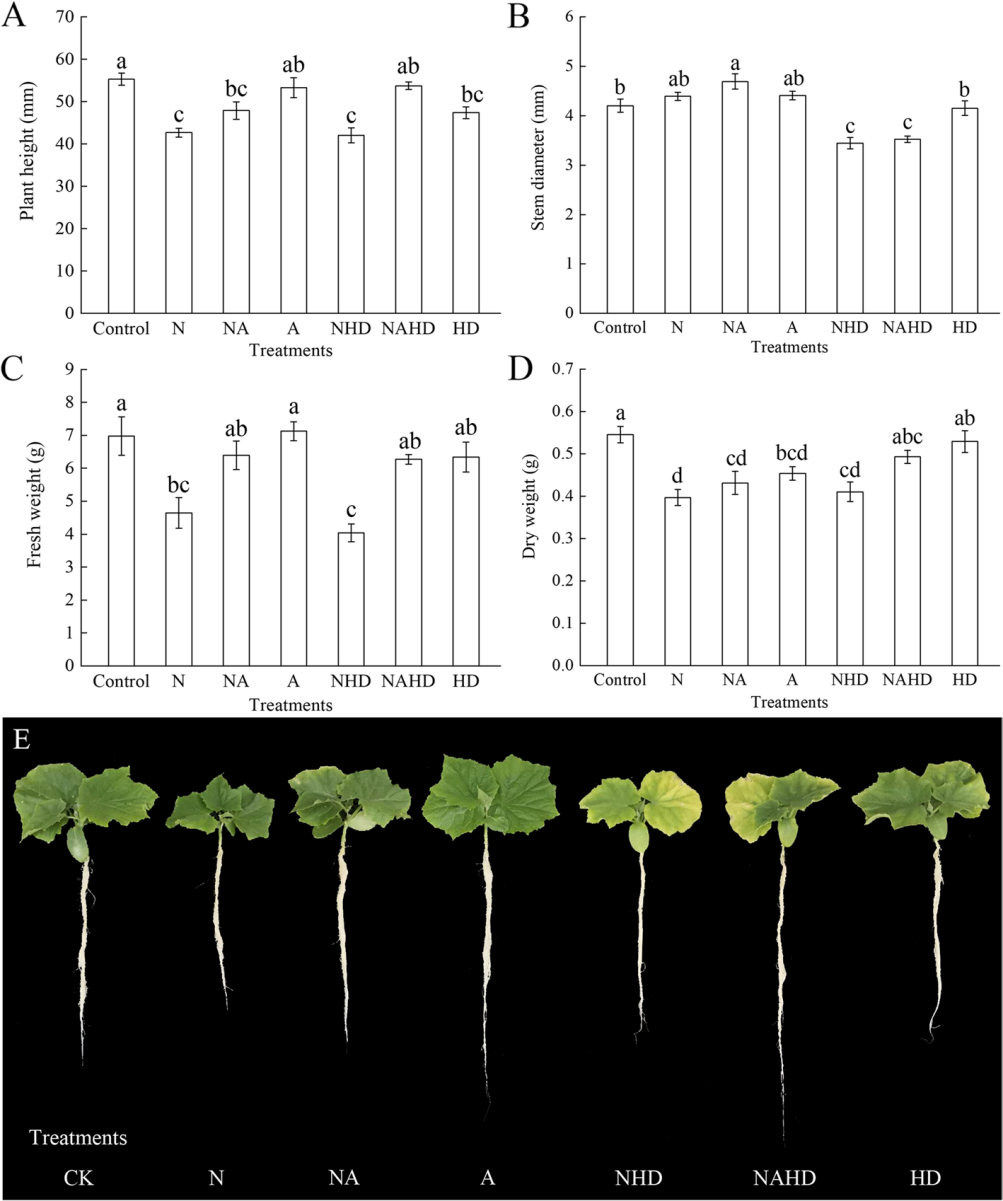
Plant height (**A**), stem diameter (**B**), fresh weight (**C**) dry weight (**D**) and seedling morphology (**E**) of cucumber seedlings. Values were mean ± SE (*n* = 5). Bars with different lowercase letters were significantly different by Tukey’s multiple range test (*P* < 0.05)

The plant height, stem diameter, fresh weight, and dry weight of cucumber seedlings are shown in Fig. 2. As compared to those in the control seedlings, NaCl stress decreased plant height, fresh weight, and dry weight by 22.8%, 33.4%, and 27.3%, respectively. These indices decreased sequentially when the seedlings were exposed to NHD treatment, while the seedling stem diameter decreased by 18.1%, as compared to that in the control. Application of 25 mg L^–1^ ALA (NA) increased the plant height, stem diameter, fresh weight, and dry weight of the cucumber seedlings slightly, as compared to those upon NaCl treatment alone. When endogenous heme was removed (NHD), the growth indices were significantly reduced compared to those in the control, while the plant height, fresh weight, and dry weight were not significantly different as compared to those upon NaCl treatment. However, the etiolation of shoots under the NHD treatment was more pronounced than that under the N treatment. Under the NAHD treatment, the sprayed ALA enhanced the plant height and fresh weight significantly, as compared to those under the NHD treatment.

### Heme is involved in the ALA-induced root development under NaCl stress

**Fig. 3:**
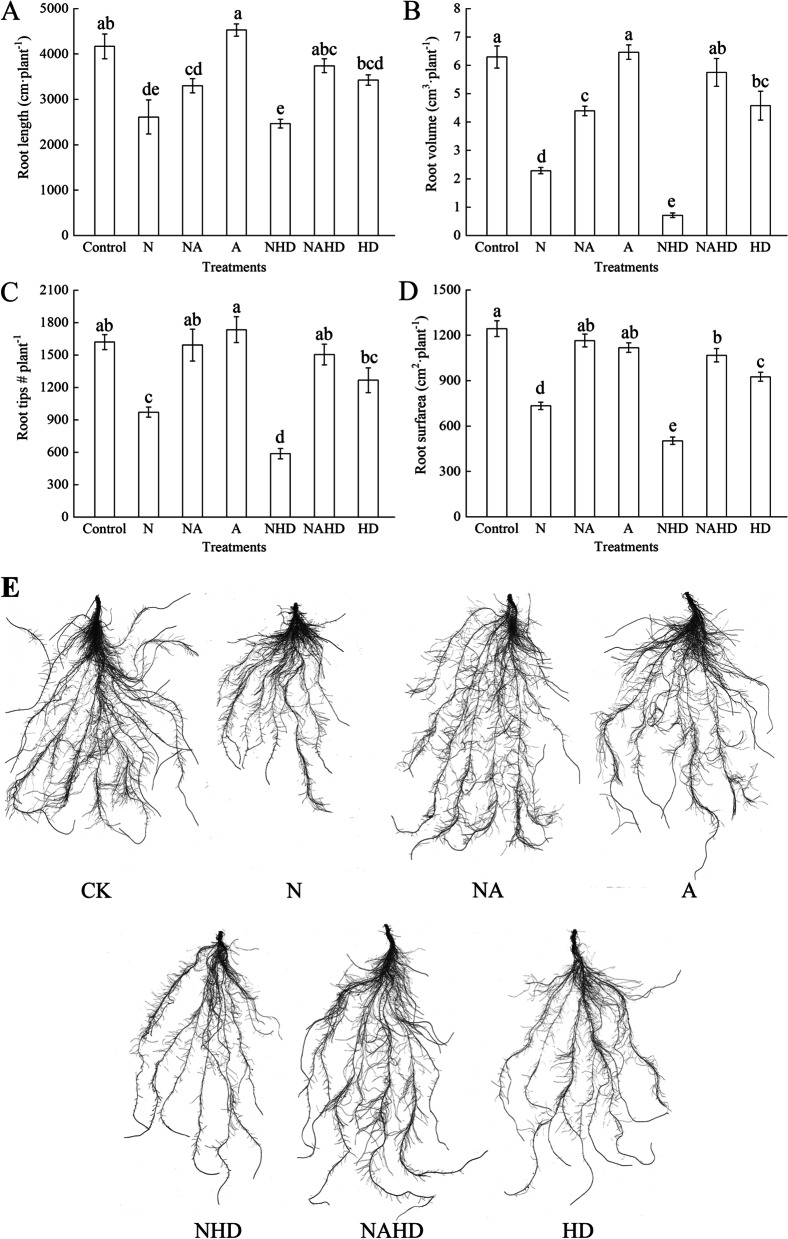
Root length (**A**), root volume (**B**), root tips (**C**), root surface area (**D**), and root morphology (**E**) of cucumber seedlings. Values were mean ± SE (*n* = 5). Bars with different lowercase letters were significantly different by Tukey’s multiple range test (*P* < 0.05)

Figure 3 shows the root indices of cucumber seedlings when the heme biosynthesis pathway was inhibited under salt stress. Compared to the control treatment, NaCl significantly decreased root length, root volume, number of root tips, and root surface area of the cucumber seedlings by 37.3%, 63.7%, 40.0%, and 41.0%, respectively. These indices were further reduced under salinity, when heme was removed (NAHD). Moreover, ALA application promoted cucumber root growth under stressful conditions (NA and NAHD). In addition, under normal culture conditions, ALA treatment alone did not affect the root indices. However, exogenous Hx and DPD inhibited root length, volume, number of root tips, and surface area.

### Heme is involved in ALA-alleviated membrane permeability and root activity under NaCl stress

**Fig. 4:**
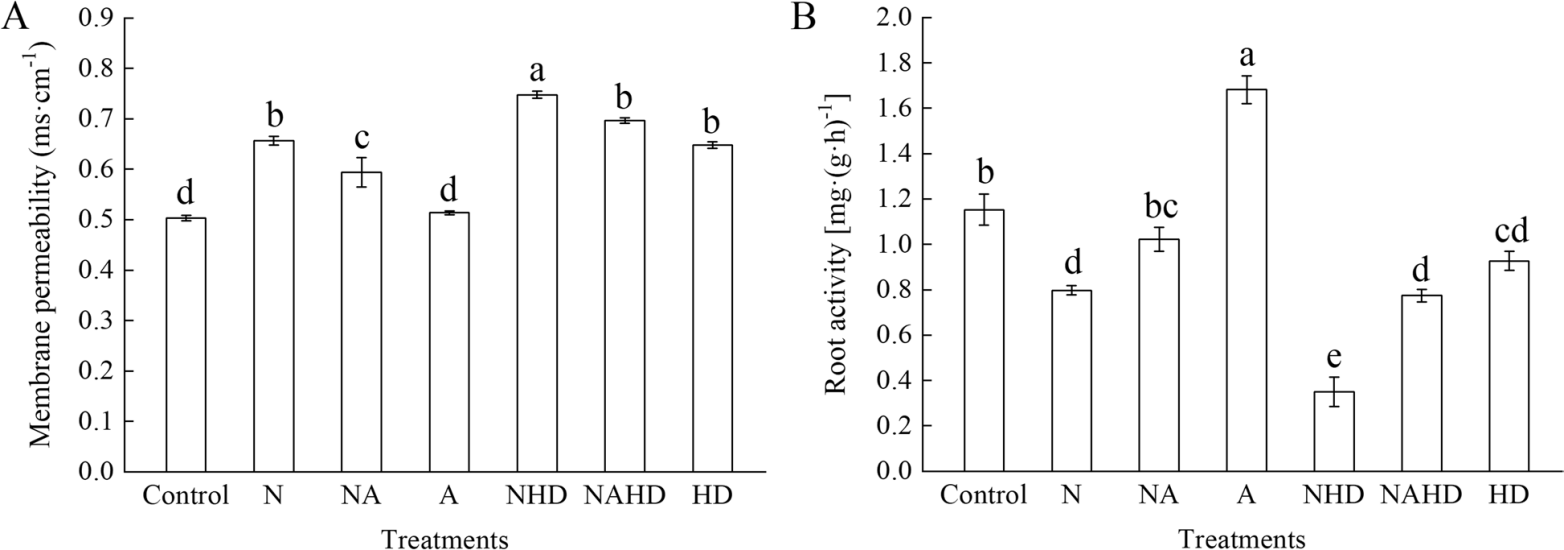
Membrane permeability (**A**) and root activities (**B**) of cucumber seedlings. Values were mean ± SE (*n* = 3). Bars with different lowercase letters were significantly different by Tukey’s multiple range test (*P* < 0.05)

NaCl stress significantly increased membrane permeability by 30.4%, as compared to that in the control (Fig. 4). Exogenous ALA effectively decreased the membrane permeability (NA) of the seedlings under salinity stress, as compared to that of the NaCl-treated seedlings. Compared to that upon NaCl treatment, the membrane permeability of leaves was significantly higher when heme was removed (NHD). Application of ALA (NAHD) significantly reduced membrane lipid peroxidation, as compared to that under the NHD treatment. On the contrary, root activity was reduced by 30.8% under NaCl stress and further reduced by 69.6% under NHD treatment, as compared to that in the control. Exogenous ALA significantly relieved the inhibition of root cells under both the NA and NAHD treatments.

### Heme is involved in the ALA-promoted anti-oxidant defense system under NaCl stress

**Fig. 5:**
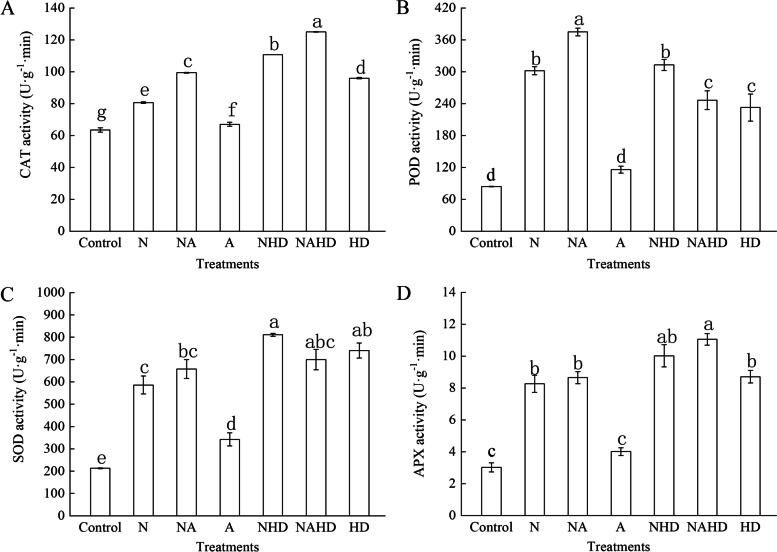
The activities of CAT (**A**), POD (**B**), SOD (**C**) and APX (**D**) of cucumber seedlings. Values were mean ± SE (*n* = 3). Bars with different lowercase letters were significantly different by Tukey’s multiple range test (*P* < 0.05)

Figure 5 shows the activities of anti-oxidant enzymes (CAT, POD, SOD, and APX) in cucumber leaves under the different treatments. The activities of these anti-oxidant enzymes were significantly higher than those of the control plants under NaCl stress. Application of ALA under salt stress (NA) caused a significant increase in the activities of CAT and POD, as compared to those upon NaCl treatment alone. In addition, as compared to those under salt stress alone, the activities of CAT, SOD, and APX were further promoted by 74.3%, 281.0%, and 231.8%, respectively, when NHD treatment was applied. Moreover, the activities of CAT and APX improved upon exogenous ALA administration under NHD treatments, while the activities of SOD and POD decreased. Furthermore, exogenous Hx and DPD dramatically enhanced the activities of anti-oxidant enzymes in the cucumber seedlings, as compared to those in the control.

### Heme is involved in the dynamic state of osmo-regulation under NaCl stress

**Fig. 6:**
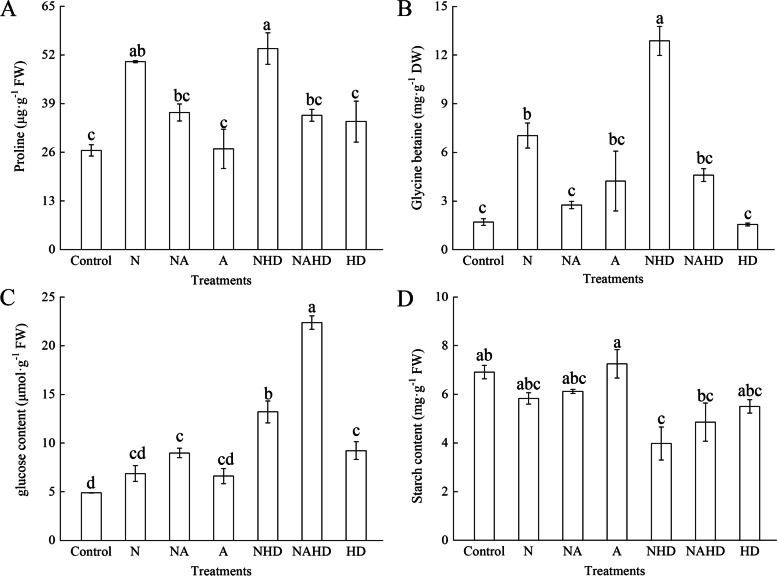
The contents of proline (**A**), glycine betaine (**B**), glucose contents (**C**) and starch contents (**D**) of cucumber seedlings. Values were mean ± SE (*n* = 3). Bars with different lowercase letters were significantly different by Tukey’s multiple range test (*P* < 0.05)

Figure 6 shows the content of osmotic adjustment substances, including proline, glycine betaine, glucose, and starch, in the cucumber seedlings under different treatments. The results showed that both the proline and glycine betaine contents increased under NaCl and NHD treatments, as compared to those in the control group. However, upon the NA and NAHD treatments, these contents decreased significantly, as compared to those under the N and NHD treatments. In addition, stressful conditions, such as the N and NHD treatments, increased the glucose content in the cucumber leaves, as compared to that in the control. The application of ALA under stressful conditions could further increase the glucose content. Moreover, as a photosynthetic product, starch content could be reduced by NaCl, and upon removal of the endogenous heme (NHD), the starch content decreased by 42.5%, as compared to that in the control. After spraying with ALA (NAHD), the starch content increased. However, there were no significant differences between the N and NA treatments.

### The contents of the derivatives of the ALA metabolic pathway could be affected by NaCl stress

**Fig. 7:**
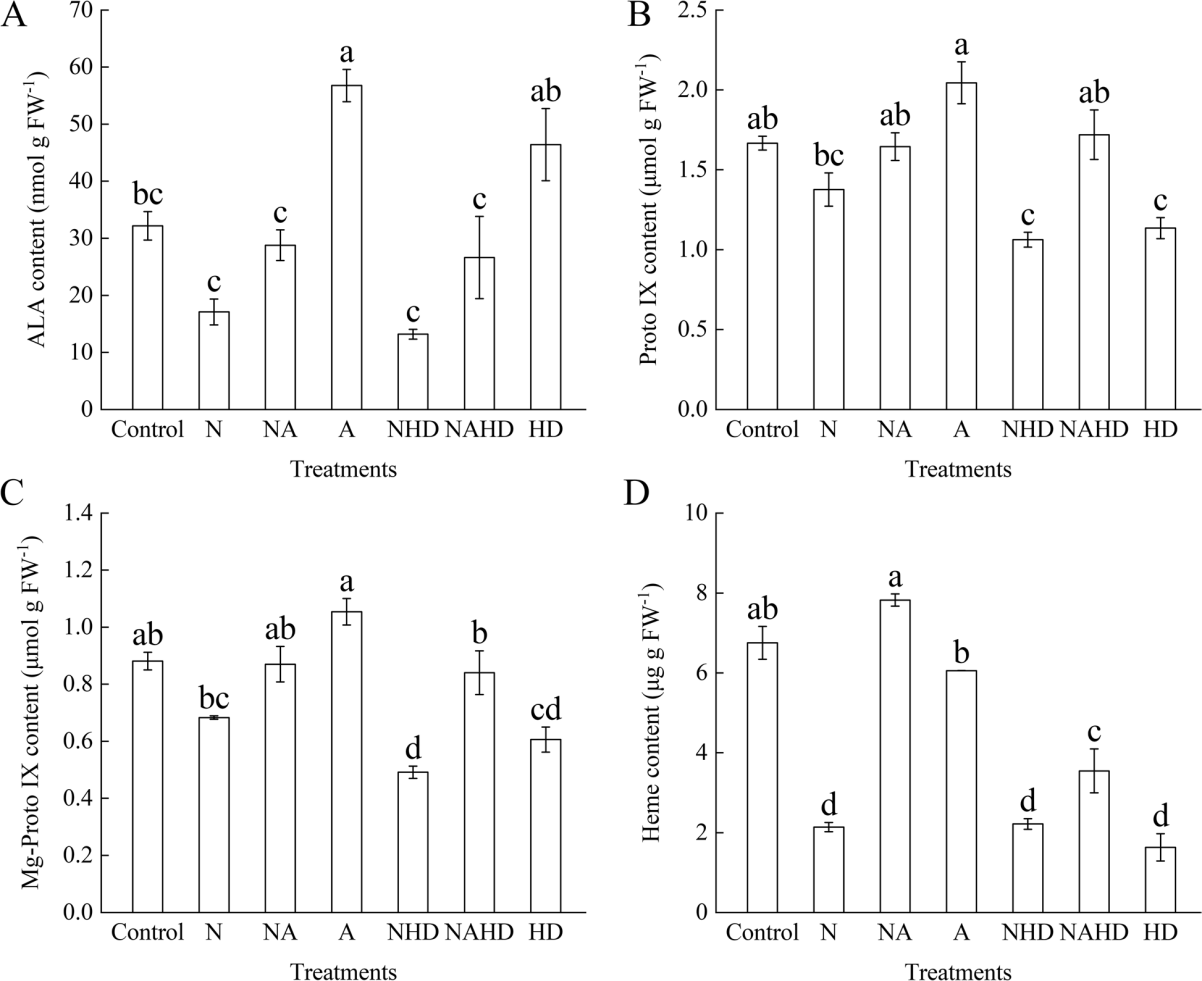
The contents of endogenous ALA (**A**), Proto IX (**B**), Mg-Proto IX (**C**) and heme (**D**) in cucumber seedlings. Values were mean ± SE (*n* = 3). Bars with different lowercase letters were significantly different by Tukey’s multiple range test (*P* < 0.05)

The contents of key derivatives, including endogenous ALA, protoporphyrin IX (Proto IX), Mg-protoporphyrin IX (Mg-Proto IX), and heme, are shown in Fig. 7. NaCl stress significantly suppressed the content of the derivatives, and upon application of Hx and DPD (NHD treatment), these contents decreased further. As compared to those in the control group, endogenous ALA decreased by 59.01%, Proto IX decreased by 36.24%, Mg-Proto IX decreased by 44.22%, and heme decreased by 67.18% under NHD treatment. The application of ALA can increase endogenous ALA content under stressful or non-stressful conditions, and contents of Proto IX and Mg-Proto IX increased. Similarly, the heme content significantly decreased under N or NHD treatment, as compared to that under the control treatment, while exogenous ALA reversed this phenomenon and increased the heme content in the cucumber leaves. Heme content increased by 265.65% under the NA treatment, as compared to that under the NaCl treatment; meanwhile, it increased by 60.04% under the NAHD treatment, as compared to that under the NHD treatment.

### The expression of the key genes involved in the ALA metabolic pathway could be regulated by NaCl stress

**Fig. 8:**
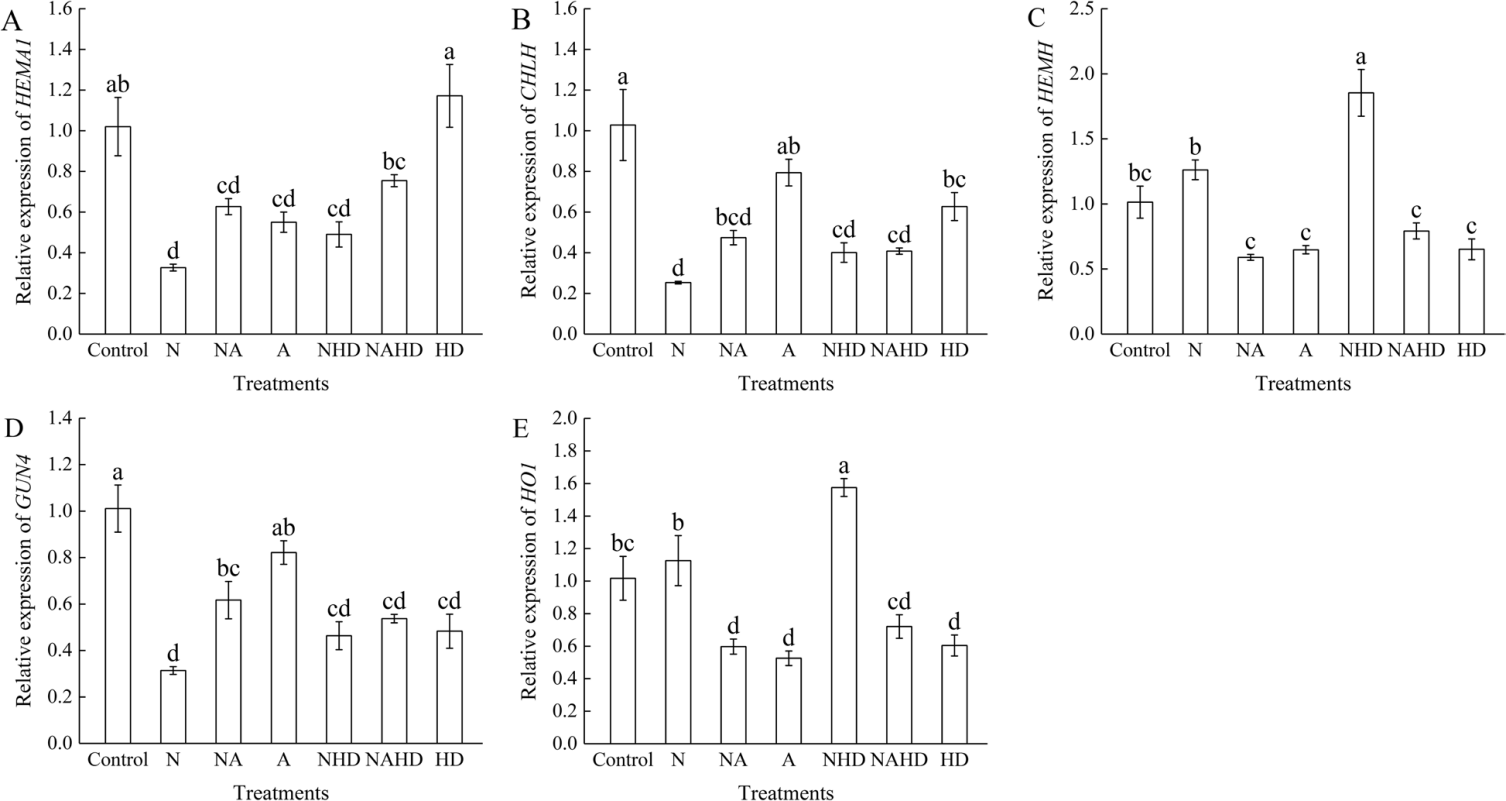
The relative expression levels of *HEMA1* (**A**), *CHLH* (**B**), *HEMH* (**C**), *GUN4* (**D**) and *HO1* (**E**) in cucumber seedlings. Values were mean ± SE (*n* = 3). Bars with different lowercase letters were significantly different by Tukey’s multiple range test (*P* < 0.05)

The relative expression levels of the key genes involved in the ALA metabolic pathway are shown in Fig. 8. The expression of *HEMA1* was inhibited by exogenous ALA and NaCl, while exogenous Hx and DPD did not affect the same. In the Mg-branch of the ALA metabolic pathway, the expression of *CHLH* and *GUN4* was downregulated under salt stress and upregulated under NA treatment. However, the application of ALA under NaCl stress, with Hx and DPD, did not affect their expression levels. In the Fe-branch of the ALA metabolic pathway, *HEMH* and *HO1* expression levels were slightly upregulated under NaCl treatment, but were not significantly different from those in the control. However, when endogenous heme was inhibited and scavenged under NaCl stress, the *HEMH* and *HO1* expression levels were upregulated. Under NAHD treatment, their expression levels were downregulated, as compared to those under NHD treatment.

## Discussion

Plant growth can be significantly inhibited in a relatively highly concentrated salt environment, owing to many physiological and biochemical process changes. Regulating plant growth and avoiding damage under environmental stress has become an important issue in the agricultural research field. In studies of exogenous plant growth regulators, ALA, an important precursor of tetrapyrrol, has increasingly been used in agricultural production, as a green non-toxic regulator. A series of studies have shown that ALA can alleviate plant injury and play a positive role in plant growth and development under abiotic stress [5]. Chen et al. showed that foliar application of 1.25 mmol L^–1^ ALA significantly alleviated salt stress in watermelon seedlings [21]. In addition, 20 mmol L^–1^ ALA significantly mitigated the damage caused to sunflower seedlings under 150 mmol L^–1^ NaCl stress [46]. Our results are consistent with those of previous studies, as we also found that plant growth was significantly limited by salt stress, while the application of ALA effectively protected the plants against damage. Moreover, under NaCl + DPD + Hx conditions, the growth of the plants was further inhibited, with severely etiolated leaves, indicating that endogenous heme may act as a responsive factor to salt stress. However, when seedlings were sprayed with ALA, the plant biomass improved significantly. It has been suggested that exogenous ALA may play an important role in helping plants adapt to stressful conditions.

Root system development in the present study was sensitive to the different treatments. As the main organs of plants that absorb nutrients and water from the soil, root growth and architecture are extremely sensitive to Na^+^ stress [47]. Therefore, the growth status of the roots can directly indicate damage under stressful soil conditions. Many studies have shown that abiotic stresses can damage plant roots in some species, such as rice [48], maize [49], *Arabidopsis* [50], and oilseed rape [51]. In this study, our results showed that NaCl stress significantly changed plant morphology, limited root growth, and reduced the number and length of the lateral roots. Moreover, under NaCl stress, DPD and Hx significantly decreased root length, volume surface area, and number of lateral roots, as compared to those under NaCl stress. The growth indices of seedlings under stressful conditions (N and NHD) increased significantly when exogenous ALA was applied. These results were in agreement with those of a study by Anwar et al. [52], which reported that exogenous ALA improves the root length of cucumber seedlings under low temperature stress. In addition, 200 mg L^–1^ ALA could increase root activity and effectively improve the physiological characteristics of peaches under salinity stress [25]. The results of the present study showed that exogenous ALA can promote root morphological parameters and root activity of plants under salt stress, and when endogenous heme is inhibited and scavenged, exogenous ALA promotes the physiological processes of plant roots under salt stress and improves plant growth. Salinity stress enhances the production and accumulation of ROS [9], resulting in membrane lipid peroxidation and cell damage [53, 54]. Previous reports have shown that exogenous ALA reduced the malondialdehyde (MDA) content in the leaves of *Isatis indigotica* seedlings [55] and the degree of membrane lipid peroxidation in peach seedlings under salt stress [25]. In our study, membrane permeability increased significantly under salt stress, leading to a reduction in root cell activities. The value of membrane permeability reached the maximum value under treatment with NaCl + Hx + DPD, revealing that the plants were under extremely severe oxidative stress. This result indicated that the inhibition and scavenging of endogenous heme caused further damage to plants when the plants were already under stress. In addition, it was indirectly revealed that heme synthesis in plants can positively regulate plant stress resistance. Furthermore, our results showed that ALA inhibited the increase in membrane permeability of cucumber roots and enhanced root cell activity. These findings were consistent with those of previous studies [56, 57]. Exogenous ALA not only reduced oxidative stress under salt stress but also improved plant growth when cucumber seedlings were exposed to Hx and DPD under salt stress. This indicates that endogenous heme is involved in the response and adaptation of plants to salinity. Anti-oxidant enzymes play an indispensable role to different degrees in the adaptation to salt stress [58]. Previous studies have shown that exogenous ALA enhances the activities of SOD, POD, CAT, and APX under salt [59] and heavy metal stress [60]. In this study, the activities of SOD, POD, CAT, and APX increased with NaCl treatment alone. CAT, SOD, and APX activities further increased upon treatment with NaCl, Hx, and DPD. This suggests that plants suffer from more severe oxidative stress under salinity conditions, when endogenous heme is removed. A previous study and our current findings demonstrated that heme is involved in the ALA-induced enhancement of the anti-oxidant defense system of plants under salt stress.

When plants are under abiotic stress, glucose and sucrose, which are important carbon assimilation products, also play important roles as osmotic regulatory substances, to balance the plant osmotic pressure [61, 62]. Salinity significantly increases the glucose and sucrose contents in plant leaves, which increase with increasing salt concentration [63]. Exogenous ALA effectively reduced glucose and sucrose content under salt stress in creeping bentgrass [23]. In the present study, the glucose content increased under NaCl stress, as compared to that in the control, while exogenous ALA further improved its content. A similar trend was observed under NHD and NAHD treatments. Moreover, the starch content decreased significantly under salt stress. These results indicated that plants can adapt to osmotic stress by degrading starch into glucose, to increase the level of osmotic adjustment substances, and exogenous ALA helps to enhance the contents of starch and glucose. These findings show that plants may increase their own metabolism of carbonized products under stress environments, as a way to improve their stress resistance, and exogenously applied ALA can contribute to maintaining this balance. Consistent with this finding, several studies suggest that soluble sugar not only provides energy and raw material supply for plant growth and metabolism but also effectively maintains the balance of plant osmotic pressure [64, 65].

Glutamate betaine [28] and proline are important signals for plant response to osmotic stress. As important osmotic regulatory substances, GB and proline can effectively regulate intracellular osmotic potential, maintain cell osmotic pressure, and reduce the damage caused by stress in plants [66–68]. These also play important roles in regulating plants response to drought and salt stresses [59, 69]. In the present study, the levels of proline and GB in cucumber seedling leaves increased under NaCl stress, consistent with the findings of previous studies. In addition, exogenous ALA decreased GB and proline accumulation in cucumber seedlings, under both NaCl and NHD treatments. ALA restrained plant damage though regulated osmotic substances [9, 70]. Interestingly, ALA and proline are important intermediate materials in the metabolic branch of their common precursor glutamate, and the mechanism of the metabolic relationship between ALA and proline has not yet been demonstrated [6]. Heme produced by the downstream metabolism of ALA can suppress the production of endogenous ALA, by inhibiting its synthase activity [71]. In the present study, we found that Hx and DPD markedly promoted the accumulation of proline and glutamate betaine in cucumber seedling leaves under stressful conditions, and exogenous ALA reversed this phenomenon. These results indicated that plants respond to salt stress by promoting the accumulation of glutamate betaine and proline. Scavenging and inhibiting endogenous heme leads to a significant increase in GB and glucose levels, suggesting that heme is involved in the salt response of higher plants. Furthermore, exogenous ALA enhanced the synthesis of heme, which contributed to resistance against damage caused by salinity.

Plant heme has been reported as a light-sensitive substance, when relatively large accumulated in plant tissue it can be motivated by light, produces ROS, and causes damage to cells [30]. There is a tryptophan-rich sensory protein (TSPO) in *Arabidopsis thaliana* that can enhance plant tolerance to oxidative stress, by binding and scavenging free heme [31]. However, in the present study, when Hx and DPD were applied to cucumber seedlings subjected to NaCl stress, there was more aggravated damage to plants, than that under NaCl stress alone. Meanwhile, under salinity, *HEMH* and *HO1* were upregulated in plant tissues, while the heme content decreased. These results indicated that the biosynthesis and metabolism of heme might be enhanced to adapt to stressful conditions. Researchers have also found that applying exogenous heme to *Arabidopsis* did not induce the production of endogenous ROS [33]. Overexpression of the ferrochelatase gene (*BjFeCh*) of rhizobia (*Bradyrhizobium japonicum*) in rice plants increased the amount of heme synthesis and enhanced the resistance of transgenic plants to herbicide stress [34]. Heme is a plastid-produced signal responsible for signal transduction from the chloroplasts to the cytoplasm, and for regulating transcription factors necessary for stress resistance [32]. Our research also suggests that heme plays a positive role in plant response to salt stress. Heme can be transformed into CO, free iron (Fe^2+^), and biliverdin, which are catalyzed by heme oxygenase (HO) [72]. It is worth mentioning that BV is physiologically an effective anti-oxidant in plants [73, 74]. In addition, when ALA was applied to plants under stress (such as NA and NAHD treatments), the heme content increased in the leaves, indicating that exogenous ALA might act as a supplement to heme synthesis. In addition, the expression of *HEMA1* was downregulated under NaCl and ALA treatment in our study. The rate-limiting enzyme Glu-tRNA reductase, encoded by *HEMA1* in this pathway, can be regulated by downstream products [27]. Under salt stress, chlorophyll biosynthesis could be inhibited and the downstream derivatives of ALA accumulate [28], as observed in the present study as well. The expression of *HEMA1* is suppressed by the negative feedback regulation of this pathway.

## Conclusions

In this study, we screened Hx (an effective heme scavenger) and DPD (an effective ferrochelatase inhibitor), for use in further plant research.

**Fig. 9:**
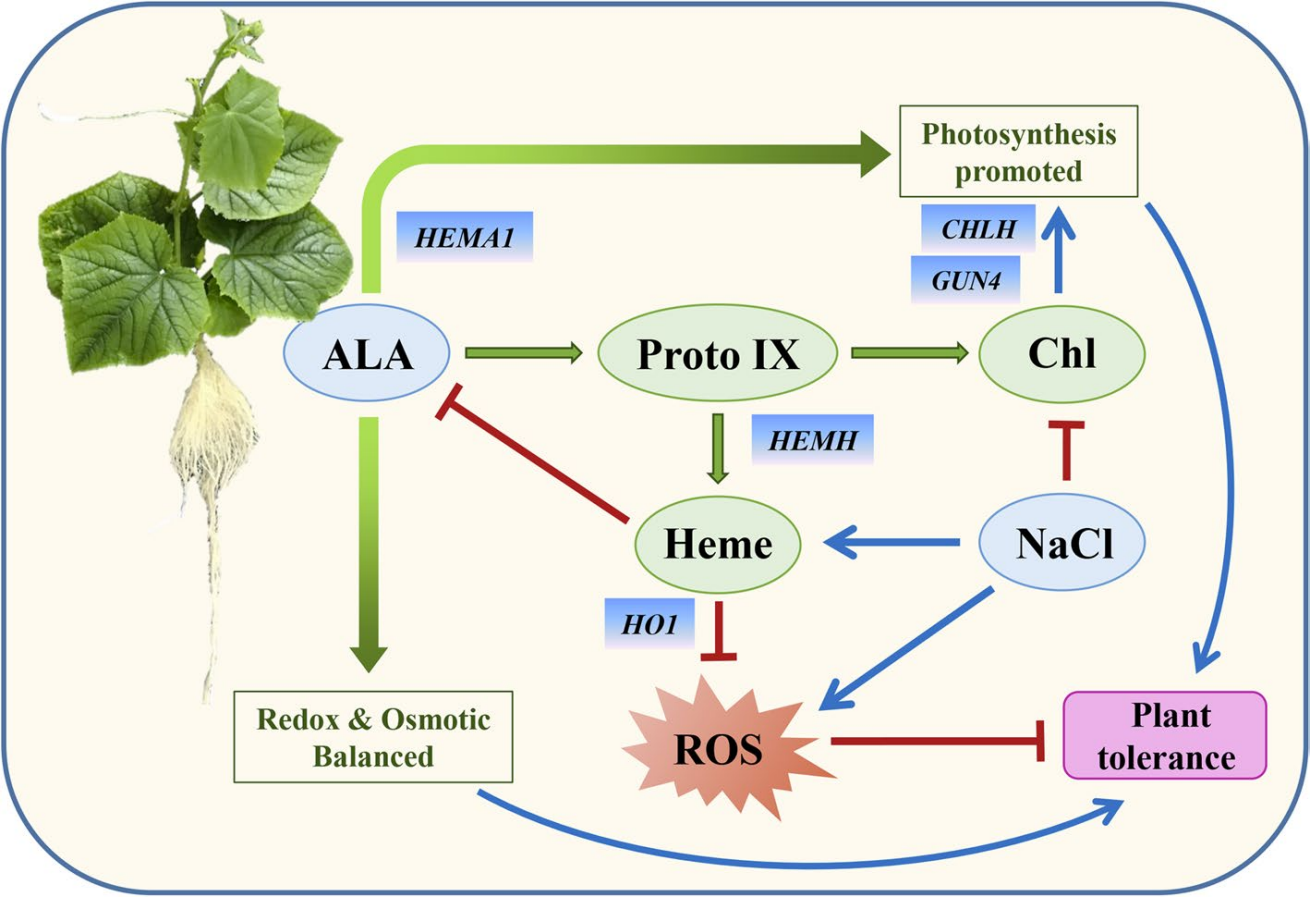
A model illustrating of heme involving in ALA-induced salinity response of cucumber seedling

Salt stress suppresses the normal growth of plants; however, the scavenging of endogenous heme exacerbated the inhibition of plant growth. Exogenous ALA significantly alleviated the growth inhibition and oxidative damage of plants caused by salinity, regulated the activity of anti-oxidant enzymes and the contents of osmotic adjustment substances, balanced the redox metabolism, and protected the stability of the photosynthetic system. Our results demonstrated that endogenous heme, as a metabolite of ALA, is positively involved in the enhancement of stress resistance induced by exogenous ALA (Fig. 9).

## Data Availability

All data generated or analyzed during this study are included in this published article.
